# A35 INTESTINAL REGENERATION IS IMPAIRED IN HIRSCHSPRUNG-ASSOCIATED ENTEROCOLITIS

**DOI:** 10.1093/jcag/gwad061.035

**Published:** 2024-02-14

**Authors:** B Li, Z Zhang, D Lee, C Lee, Q Jiang, A Pierro

**Affiliations:** The Hospital for Sick Children, Toronto, ON, Canada; The Hospital for Sick Children, Toronto, ON, Canada; The Hospital for Sick Children, Toronto, ON, Canada; The Hospital for Sick Children, Toronto, ON, Canada; The Hospital for Sick Children, Toronto, ON, Canada; The Hospital for Sick Children, Toronto, ON, Canada

## Abstract

NOT PUBLISHED AT AUTHOR’S REQUEST

Please acknowledge all funding agencies listed below.

Funding Agencies

NFRF

